# Long non-coding RNAs as the critical regulators of doxorubicin resistance in tumor cells

**DOI:** 10.1186/s11658-021-00282-9

**Published:** 2021-08-23

**Authors:** Ghazaleh Khalili-Tanha, Meysam Moghbeli

**Affiliations:** Department of Medical Genetics and Molecular Medicine, School of Medicine, Mashhad University of Medical Sciences, Mashhad, Iran

**Keywords:** Doxorubicin, Drug resistance, Cancer, Chemotherapy

## Abstract

Resistance against conventional chemotherapeutic agents is one of the main reasons for tumor relapse and poor clinical outcomes in cancer patients. Various mechanisms are associated with drug resistance, including drug efflux, cell cycle, DNA repair and apoptosis. Doxorubicin (DOX) is a widely used first-line anti-cancer drug that functions as a DNA topoisomerase II inhibitor. However, DOX resistance has emerged as a large hurdle in efficient tumor therapy. Furthermore, despite its wide clinical application, DOX is a double-edged sword: it can damage normal tissues and affect the quality of patients’ lives during and after treatment. It is essential to clarify the molecular basis of DOX resistance to support the development of novel therapeutic modalities with fewer and/or lower-impact side effects in cancer patients. Long non-coding RNAs (lncRNAs) have critical roles in the drug resistance of various tumors. In this review, we summarize the state of knowledge on all the lncRNAs associated with DOX resistance. The majority are involved in promoting DOX resistance. This review paves the way to introducing an lncRNA panel marker for the prediction of the DOX response and clinical outcomes for cancer patients.

## Background

Chemotherapy is an effective method of tumor therapy, but some tumors cannot be treated effectively due to multidrug resistance (MDR) [1, 2]. A chemotherapeutic failure of about 85–90% has been reported for solid tumors [3], making this the main reason for tumor relapse, metastasis and poor clinical outcomes for patients. Various molecular and cellular processes, including membrane transporters, oncogenes, tumor suppressors, DNA repair, apoptosis and epithelial–mesenchymal transition (EMT), are associated with chemoresistance in tumor cells [4]. In addition to this challenge, chemotherapeutic drugs can themselves cause severe side effects in patients, including cardiomyopathy, typhlitis and acute myelotoxicity [5, 6].

Doxorubicin (DOX; brand name Adriamycin) is an anthracycline that is widely used as an anticancer agent for various tumors. It inhibits DNA replication and transcription by causing DNA damage that prevents mitosis in tumor cells. It also promotes apoptosis by stimulating topoisomerase II to cut DNA strands. However, despite its wide clinical application, DOX is a double-edged sword: it damages normal tissues, thus negatively affecting the quality of patients’ lives during and after treatment. It has toxic effects on normal heart, brain, kidney and liver tissues [7].

Clarifying the molecular basis of DOX resistance could enable the development and introduction of novel therapeutic modalities with fewer and/or lower-impact side effects in cancer patients. Various genetic mutation and epigenetic mechanisms can be related with DOX resistance. Mutations in ABC transporter family members such as ABCB1 [8], ABCBG2 [9] and MRP1 [10], as well as DNA repair factors such as p53 [11–14] are considered to be the major causes of DOX resistance. There is also evidence for a role of epigenetic aberration in chemoresistance [15–17].

Long non-coding RNAs (lncRNAs) are involved in various cellular processes via transcriptional regulation of their target genes. They can also function as oncogenes or tumor suppressors [18, 19]. Based on their biogenesis, lncRNAs are categorized as intergenic, antisense, intronic, overlapping or full lapping [18]. Antisense (AS) lncRNAs are the largest category, making up about 70% of the long non-coding transcriptome [20].

**Fig. 1:**
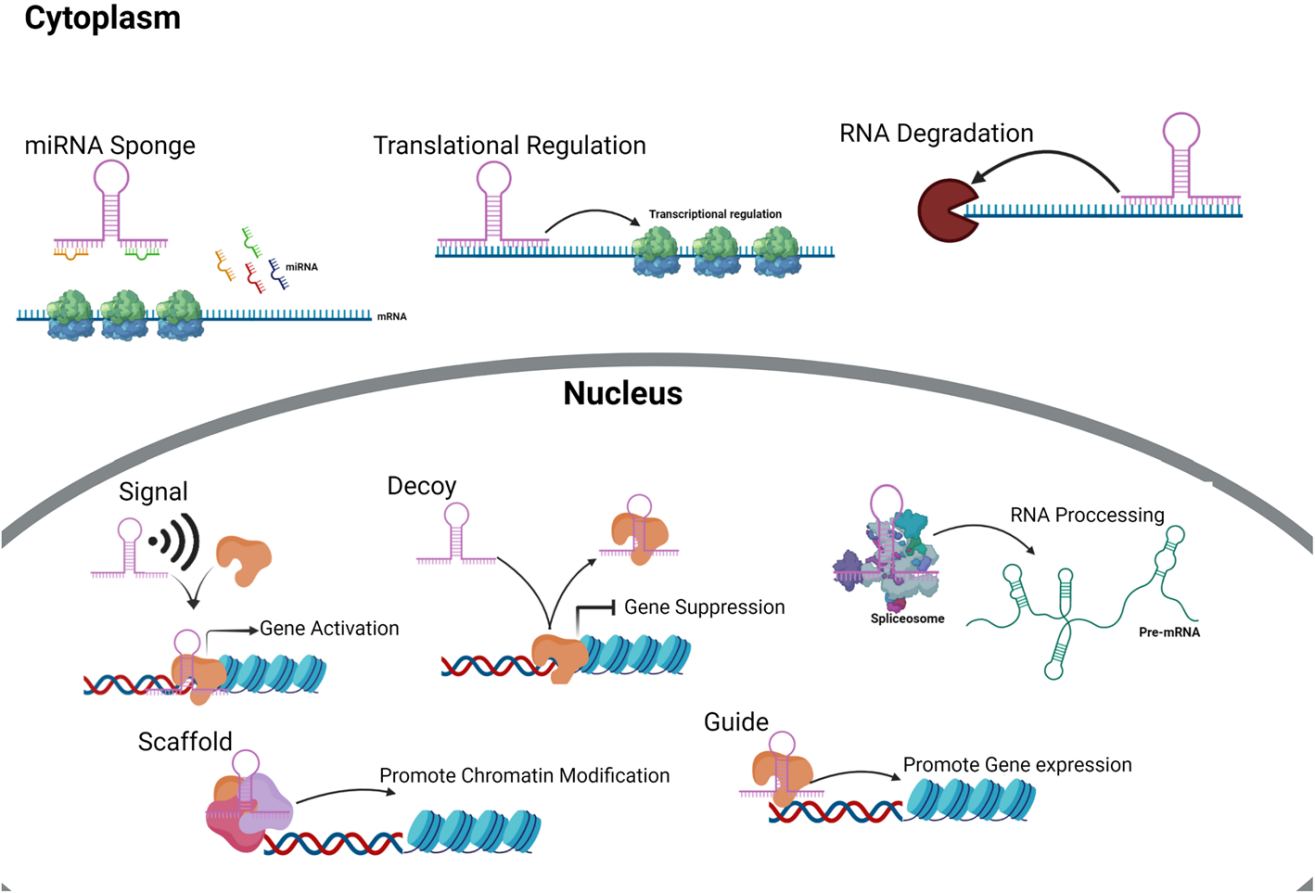
The molecular mechanisms of lncRNA actions in pathophysiological processes

**Fig. 2:**
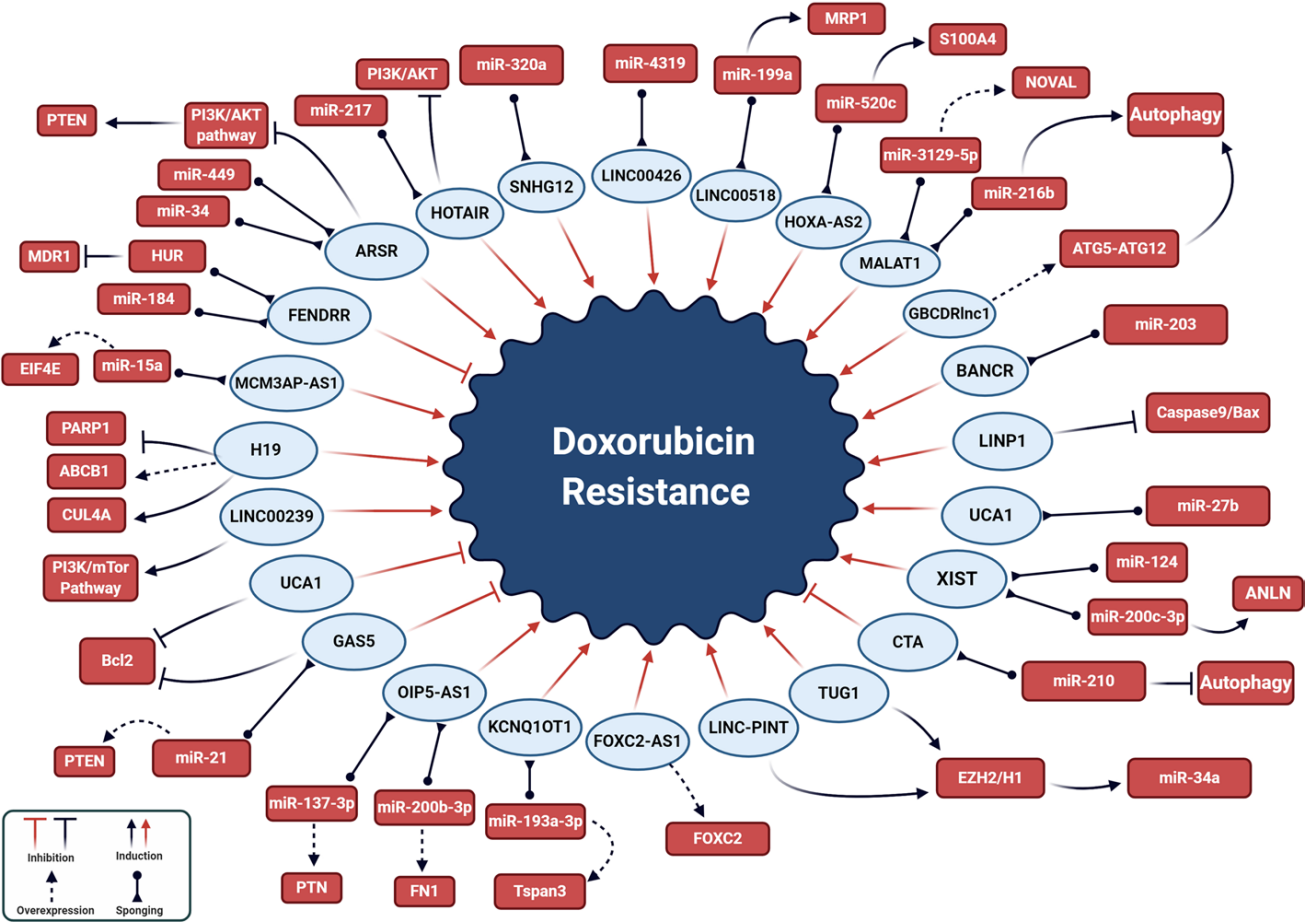
The molecular mechanisms for lncRNA regulation of doxorubicin (DOX) resistance

LncRNAs have important effects on tumorigenesis through their modulation of various pathophysiological processes, including the stability of mRNA, RNA splicing, chromatin remodeling and miRNA sponging (Fig. 1) [21–26]. Their deregulation is one of the main obstacles for the effectiveness of chemotherapy [27–29]. They are involved in chemotherapeutic responses through their regulation of histone modification and DNA methylation. Since epigenetic signatures are inheritable and reversible, they have been suggested as effective biomarkers for the prediction of chemotherapeutic outcomes [29]. This review summarizes the molecular mechanisms whereby lncRNAs affect DOX responses in tumor cells (Fig. 2, Table 1).

**Table 1:** All of the long non-coding RNAs associated with Doxorubicin response in different cancers

Cancer type	DOX response	LncRNA	Target	Samples	Function	References
Breast cancer (BC)						
BC	Resistance	*XIST*	*miR-200c-3p /ANLN*	MDA-MB-231/ADM and MDA-MB-231 cell lines	*XIST* up regulated *ANLN* by sponging *miR-200c-3p* and inhibited cell proliferation as well as promoted apoptosis	[34]
BC	Resistance	*Linc00152*	-	40 NT* MDA-MB-231 and MCF-7 cell lines	Knockdown of *Linc00152* suppressed tumor growth, cell migration, invasion, and chemo-resistance	[41]
BC	Resistance	*Linc00518*	*miR-199a/MRP1*	30 NT MCF-10A, MCF-7/ADR and MCF-7 cell lines	*Linc00518* Knockdown suppressed *MRP1* expression and induced cell apoptosis	[44]
BC	Resistance	*HOTAIR*	PI3K/AKT	MCF-7 and DOXR-MCF-7 cell lines	*HOTAIR* suppressed PI3K/AKT pathway, reduced cell survival and promoted apoptosis	[48]
BC	Resistance	*Linc00668*	*SND1*	HMEC-hTERT, MCF-10A, MCF-7, T47D, MDA-MB-231, HS578t, and 293T, SUM149, and SUM159 cell lines	*Linc00668* interacted with SND1 and regulated SMAD2/3/4 expression, and also decreased invasion, self-renewal, and chemo-resistance	[54]
BC	Resistance	*DCST1-AS1*	*ANXA1*	MDA-MB-231, BT-549, T-47D, and MCF7 cell lines	*DCST1-AS1* targeted ANXA1 and induced EMT	[61]
BC	Resistance	*LINC00160*	*C/EBPβ/TFF3*	47 NT MCF‐7, MCF‐7/Tax, BT474, BT474/Dox and MCF10A cell lines	*LINC00160* knockdown reduced cell migration and invasion	[64]
BC	Resistance	*LINP1*	*CASP9/BAX*	MDA-MB-231, MDA-MB-231/5FU, MDA-MB-231/DOX, MDA-MB-468 and MCF7 cell lines	*LINP1* knockdown suppressed tumor growth and metastasis as well as promoted apoptosis	[65]
BC	Resistance	*H19*	*CUL4A /ABCB1/MDR1*	MCF-7 cell lines	*H19* up regulated *CUL4A* and ABCB1/MDR1 genes	[69]
BC	Resistance	*H19*	*PARP-1*	63 NT MCF-7 and MCF-7/Dox cell lines	Knockdown of *H19* increased *PARP-1* expression and induced cell death	[71]
Osteosarcoma (OS)						
OS	Resistance	*TUG1*	*AKT*	Saos-2 and MG-63 cell line	Polydatin inhibited TUG1/AKT axis and proliferation and promoted apoptosis	[83]
OS	Resistance	*FOXC2-AS1*	*FOXC2*	68 NT MG63, SaoS2 and HOS cell lines	*FOXC2-AS1* facilities *ABCB1* expression by increasing FOXC2 expression	[86]
OS	Resistance	*FOXC2-AS1*	*ABCB1*	MG63, SaoS2 and U-2OS cell lines	Silencing of *FOXC2-AS1* and ABCB1 repressed tumor growth	[89]
OS	Resistance	*OIP5-AS1*	*miR-137-3p*	56 tumor tissues and 16 normal tissues hFOB1.19, MG63, and MG63/DOX cell lines	*OIP5-AS1* knockdown inhibited proliferation and metastasis	[93]
OS	Resistance	OIP5‐AS1	*miR‐200b‐3p*	80 patients MG63, KHOS and U2OS cell lines	*OIP5‐AS1* sponged *miR‐200b‐3p* and regulated FN1 expression. Overexpression of FN1 contributed to the sensitivity of OS cells to doxorubicin	[97]
OS	Resistance	*SNHG12*	*miR-320a / MCL1*	32 doxorubicin-resistant patients and 32 doxorubicin-sensitive patients MG-63, U2OS, HOS, SAOS-2 and hFOB cell lines	*SNHG12* modulated Wnt/β-catenin pathway, so inhibited *miR-320a* expression and promoted MCL1 expression	[103]
OS	Resistance	*LINC00426*	*miR-4319*	MG63, KHOS, U2OS, MG63/DXR, and KHOS/DXR cell lines	Knockdown of *LINC00426* significantly decreased cell viability and proliferation	[104]
OS	Sensitivity	*CTA*	*miR-210*	30 patients Saos-2, U-2OS, MG-63 and MG-63/DOX cell lines	Overexpression of *CTA* reduced autophagy and promoted apoptosis	[105]
OS	Sensitivity	*FENDRR*	*ABCB1/ ABCC1*	80 patients MG63, SaoS2, HOS and MG63/DXR cell lines	*FENDRR* down regulated ABCB1 and ABCC1 as well as suppressed DOX resistance and induced cells apoptosis	[108]
Gastric cancer (GC)						
GC	Resistance	*HOTAIR*	*miR-217*	30 NT BGC-823, SGC-7901, KATO-3, MGC-803, and GES1	Knockdown of *HOTAIR* inhibited cell proliferation and migration	[116]
GC	Sensitivity	*UCA1*	*PARP*	77 NT GES-1, BGC-823 and SGC7901 cell lines	Knockdown of *UCA1* caused repression of proliferation in cancerous cells	[120]
GC	Resistance	*UCA1*	*miR-27b*	28 patients SGC-7901, SGC-7901/ADR, SGC-7901/DDP and SGC-7901/FU	Knockdown of *UCA1* induced the expression of *miR-27b*, resulting in reduction of Bcl2 expression and promotion of CASP3 expression	[123]
GC	Resistance	*D63785*	*miR-422a*	21 patients GES-1, SGC7901, MGC803, BGC823, NCI-N87, HEK293 and HEK293T cell lines	Reduced *lncR-D63785* expression repressed proliferation, invasion, and metastasis	[128]
GC	Resistance	*NEAT1*	*–*	76 NT SGC790, GES-1, SGC7901/ADR cell lines	*NEAT1* repressed cell proliferation, apoptosis, and invasion	[131]
GC	Resistance	*MRUL*	*P-gp*	SGC7901/ADR, SGC7901/VCR, SGC7901/ADR, and SGC7901 cell lines	*MRUL* depletion induced apoptosis	[132]
Leukemia and lymphoma						
AML	Resistance	*KCNQ1OT1*	*miR-193a3p / Tspan3*	74 patients and 37 healthy subjects HS-5, HL60, HL60/ADR, K562, and K562/ADR cell lines	*KCNQ1OT1* knockdown suppressed the cell proliferation and invasion	[139]
AML	Resistance	*TUG1*	*miR-34a*	36 patients and 23 healthy subject HS-5, HL60, and HL60/ADR	*TUG1* knockdown overcame ADR resistance of AML by epigenetically enhancing *miR-34a* expression	[141]
AML	Resistance	*HOXA-AS2*	*miR-520c-3p / S100A4*	48 patients U937, U937/ADR, THP-1, and THP-1/ADR cell lines	*HOXA-AS2* acted as ceRNA of *miR-520c-3p* and induced S100A4 expression. Knockdown of *HOXA-AS2* expression significantly suppressed cell proliferation	[144]
AML	Resistance	*Linc00239*	*PI3K/ATK/mTOR*	HL-60 and KG-1 cell lines	*Linc00239* activated PI3K/ATK/mTOR pathway. *Linc00239* knockdown suppressed the cell proliferation and migration	[147]
CML	Sensitivity	*FENDRR*	*HuR*	K562 and KCL22 cell lines	*FENDER* overexpression promoted cell apoptosis and suppressed cell proliferation	[155]
BL	Resistance	*MCM3AP-AS1*	*miR-15a/EIF4E*	41 patients B-NHL cell line	*MCM3AP-AS1* knockdown decreased cell viability and increased apoptosis	[160]
Hepatocellular carcinoma (HCC)						
HCC	Resistance	*MALAT1*	*miR-216b*	BEL-7402 and BEL-7402/5-FU cell lines	*MALAT1* knockdown decreased proliferation and migration	[169]
HCC	Resistance	*lncARSR*	*miR-34/ miR-449/ PTEN*	92 NT SMMC-7721 and HepG2 cell lines	*lncARSR* promoted PTEN mRNA degradation and modulated PTEN-PI3K/Akt pathway	[177]
HCC	Resistance	*MALAT1*	*miR-3129-5p / Nova1*	36 patients Huh-7 and Hep3B cell lines	*MALAT1* knockdown suppressed proliferation, migration, invasion, and promoted apoptosis	[174]
HCC	Resistance	*NEAT1*	*–*	HepG2, PLC/PRF/5, and Huh7 cell lines	*NEAT1* up regulation in DOX resistant HCC cells	[175]
HCC	Sensitivity	*GAS5*	*miR-21/PTEN*	HepG2 and HepB3 cell lines	*GAS5* regulated PTEN expression through binding to *miR-21* and reduced cell proliferation	[178]
HCC	Sensitivity	*H19*	*–*	32 NT	*H19* inhibited HCC cell proliferation following the doxorubicin treatments	[179]
Colorectal cancer (CRC)						
CRC	Resistance	*XIST*	*miR-124*	31 patients HCT116 and LoVo cell lines	*XIST* inhibited *miR-124* expression through sponging. *XIST* knockdown enhanced the anti-tumor effect of DOX	[187]
CRC	Resistance	*BANCR*	*miR-203*	32 NT HCT116, LoVo, NCM460 and HEK293T cell lines	*BANCR* knockdown suppressed tumor growth	[191]
CRC	Resistance	*GASS*	*NODAL*	HCT116 cell line	*GASS* knockdown suppressed proliferation of cancer stem cells	[194]
Thyroid and gallbladder cancers						
ATC	Sensitivity	*PTCSC3*	*STAT3/ INO80*	20 FTC tissues and 20 ATC tissues 8505C, FTC 238, and FTC 133 cell lines	*PTCSC3* regulated STAT3/ INO80 pathway and inhibited drug resistance	[203]
GBC	Resistance	*GBCDRlnc1*	*ATG5-ATG12*	45 NT NOZ and GBC-SD cell lines	*GBCDRlnc1* knockdown inhibited autophagy	[205]
Prostate and urothelial cancers						
RCC	Resistance	*LINC-PINT*	*EZH1/ EZH2*	98 tumor tissues and 16 healthy tissues HKC, 786-O, A498, 769P, Caki-2, Caki-1, ACHN, OS-RC-2, and SN12-PM6 cell lines	*LINC-PINT* knockdown decreased proliferation, cell progression, and promoted apoptosis	[208]
BCa	Sensitivity	*GAS5*	*BCL2*	82 tumor tissues and 37 healthy tissues BTCC T24, J82, CCC-HB-2, and T24/DOX cell lines	*GAS5* knockdown increased BCL2 expression and apoptosis	[212]
TCC	Resistance	*HOTAIR*	–	35 TCC tissues and 16 healthy tissues TCC T24, J82, and SV-HUC-1 cell lines	*HOTAIR* knockdown inhibited cell proliferation and promoted apoptosis	[213]
PCA	Resistance	*LOXL1-AS1*	*miR-let-7a-5p*	DU-145 and DU-145/DOX cell lines	*LOXL1-AS1* knockdown inhibited cell proliferation and migration as well as promoted apoptosis	[219]

*Normal (N) and Tumor (T) tissues

### Breast cancer

Various screening and therapeutic methods have been used against breast cancer [30], but in advanced stages, many patients still develop invasive carcinoma and have poor prognosis [31]. Although Doxorubicin is one of the most efficient drugs for breast cancer treatment, drug resistance can be observed after several treatments [32]. About 30% of breast cancer patients who receive chemotherapy experienced the poor prognosis that is associated with the expression of multidrug resistance proteins [33].

It has been reported that *XIST* increases tumor cells proliferation and suppresses apoptosis in DOX-treated MDA-MB-231 cells through upregulation of the anillin actin-binding protein (ANLN). *XIST* was suggested to be a competitive endogenous RNA that increases the levels of ANLN expression via *miR-200c-3p* targeting [34].

*Linc00152* is a critical factor during the progression of various cancers, including lung, liver, and colorectal cancer (CRC) [35–37]. Mitosis and the cell cycle could be modulated by *Linc00152* in Hela cells [38]. During EMT, epithelial tumor cells gain mesenchymal properties through reduced adhesion and increased motility. This process is involved in early stages of tumor metastasis [39, 40]. *Linc00152* upregulation has been reported in breast cancer tissues and cell lines, where it increased the levels of cell growth, migration, EMT and DOX-resistance [41].

Multidrug resistance protein 1 (MRP1) is a member of the ATP-binding cassette (ABC) C superfamily, which is involved in MDR of different tumors [42, 43]. Increased *linc00518* and *MRP1* expression levels have been reported in breast cancer tissue and cell lines. Higher expressions of *linc00518* and MRP1 were also observed in MDR breast tumor cells (MCF-7/DOX) in comparison with parental cells (MCF-7). Drug resistance could be improved through regulation of the miR-199a/MRP1 axis in breast cancer tissue. *Linc00518* upregulated the *MRP1* via *MiR-199a* sponging. The resistance of the MCF-7/DOX cell line was also increased toward DOX, VCR, and PTX treatments via the miR-199a/MRP1 axis [44].

AKT is a Ser/Thr kinase involved in cell proliferation, apoptosis, and migration. It inhibits BAD pro-apoptotic factor via phosphorylation, which results in disassembly from BCL-2/BCL-X. AKT also upregulates the pro-survival genes via NF-κB activation. There are significant correlations between increased levels of AKT1 expression and resistance toward paclitaxel [45]. The PI3K/AKT/mTOR pathway has a pivotal regulatory role in the cell cycle, cell proliferation, metabolism, and protein synthesis [46, 47]. It has been reported that *HOTAIR* promoted DOX sensitivity via repression of the PI3K/AKT/mTOR axis. Inhibition of *HOTAIR* markedly decreased the expression of MDR proteins, which resulted in reduced cell survival and the promotion of apoptosis in DOXR-MCF-7 cells. Moreover, the CASP3, BCL-2, and BAX expression levels were significantly altered following *HOTAIR* inhibition, which increased apoptosis in DOXR-MCF-7 cells [48].

Breast cancer stem cells are a sub-population of tumor cells that have the ability of self-renewal, EMT, and chemoresistance [49]. Since SND1 is able to bind with other proteins and nucleic acids, it can regulate various proteins, including transcription factors and co-regulatory factors [50]. STAT6, STAT5, and c-MYB are SND1-associated cofactor proteins [51]. SND1 is also involved in splicing through its Tudor-SN domain [52], and mRNA stabilization through staphylococcal nuclease-like domains [53]. It has been reported that there is a correlation between *linc00668* upregulation and lymph node metastasis in BC patients. *Linc00668* induces cell invasion, self-renewal properties, and DOX resistance in BC cells through SND1 binding to upregulate SMAD2/3/4 [54].

The most significant signs of the EMT process are vimentin upregulation and E-cadherin downregulation [55]. There is a negative correlation between the E-cadherin expression and tumor progression in breast cancer patients [56]. EMT progression is regulated by SNAI1, which is an EMT-specific transcription factor that represses E-cadherin expression and promotes tumor invasion [57]. Vimentin is a type III intermediate filament produced by fibroblasts and endothelial cells. Tumor invasion can be decreased through vimentin downregulation as a consequence of re-epithelialized cells [58]. Annexin A1 (ANXA1) is in the calcium-dependent phospholipid-binding protein family involved in anti-inflammation [59]. It has also pivotal roles in the regulation of cell proliferation, adhesion, and metastasis [60]. The canonical TGF-β signaling pathway modulates EMT. Moreover, TGF-β can induce EMT via non-canonical pathways, including the ERK1/2, GTPase, and p38 MAPK pathways [55]. It has been reported that *DCST1-AS1* increases TGF-β-induced EMT and DOX resistance via ANXA1 targeting in breast cancer cells. *DCST1-AS1* inhibition also regulates TGF-β-induced production of MMP2 and MMP9 [61].

LncRNAs have a critical role in the chemoresistance of breast tumor cells through interactions with transcription factors. C/EBPβ is a transcription factor regulated by *LINC00160*, which targets TFF3. C/EBPβ is associated with a poor prognosis in estrogen receptor-negative and metastatic mammary tumors [62]. TFF3 is more highly expressed in metastatic breast cancer than in the non-metastatic type [63]. *LINC00160* is associated with paclitaxel resistance and DOX resistance in MCF-7 and BT474 cells, respectively. Overexpression of *LINC00160* is correlated with poor overall survival in BC tissues. *LINC00160* upregulated TFF3 via C/EBPβ, which resulted in DOX-resistance in BT474 cells [64].

LncRNA in non-homologous end-joining pathway 1 (LINP1) is an oncogene that suppresses tumor growth and metastasis. LINP1 upregulation has a positive association with drug-resistance and unfavorable prognosis, and is seen in breast cancer cells resistant to 5-FU and doxorubicin. It has been reported that LINP1 regulates the cell cycle via CDK4, CCND1 and CCND3 modulations. LINP1 suppresses apoptosis and induces EMT process. There is a negative correlation between P53 and LINP1. The 5-FU and DOX resistance of breast cancer cells are increased by LINP1. LINP1 represses CASP9/BAX and CASP8/9 expressions induced by 5-FU and DOX, respectively. There is also a correlation between the levels of LINP1 expression and tumor metastasis and stage [65].

*H19* is an imprinted lncRNA that is only active when inherited maternally. *H19* imprinting is regulated by a cis-acting upstream sequence that is involved in the regulation of DNA methylation and replication of parental chromosomes [66]. *H19* has a pivotal role during tumorigenesis: its upregulation is observed in about 70% of breast cancer patients [67, 68]. It has been reported that there is a significant *H19* upregulation in DOX-resistant BC cells. *H19* regulates DOX-resistance through upregulation of CUL4A and ABCB1/MDR1 [69]. Poly (ADP-ribose) polymerase (PARP) is involved in the detection of DNA damage. It employs DNA repair proteins through ADP-ribose binding. It is also involved in cell cycle and transcriptional regulations [70]. It has been reported that there is a significant *H19* upregulation in BC tissues compared with their normal margins. There is also significant *H19* upregulation in DOX-resistant tissues and cell lines. *H19* increases DOX-resistance via PARP-1 targeting in breast tumor cells [71].

### Osteosarcoma

Osteosarcoma (OS) is the most frequent bone tumor among adolescents and children, accounting for up to 20% of bone malignancies. Cisplatin, doxorubicin, or methotrexate is considered to be the standard treatment methods for advanced osteosarcoma. However, 40–45% of osteosarcoma patients are resistant toward doxorubicin treatment [72]. Taurine upregulated gene 1 (TUG1) is an oncogenic lncRNA that is associated with chemoresistance in various cancers [73, 74]. *TUG1* functions in post-transcriptional regulation through miRNA sponging and interacting with PRC2 complex [75]. *TUG1* recruits EZH2 to downregulate CDK inhibitors such as p16 and p21 in gastric carcinoma (GC) [76]. It is also involved in tumor cell proliferation and migration through regulation of the Hedgehog, PI3K/AKT, and WNT signaling pathways in HCC and OS cells [77, 78]. Polydatin is a stilbenoid glucoside isolated from some plants that is involved in cell proliferation inhibition and apoptosis induction [79, 80]. AKT phosphorylation is critical for cell survival. *TUG1* promotes osteosarcoma proliferation and invasion via AKT activation. In a positive feedback, AKT also upregulates *TUG1* [81]. Polydatin inhibits tumor cells through suppression of the PI3K/AKT and PDGF/AKT pathways [81, 82]. It has been reported that polydatin inhibits osteosarcoma cell proliferation and reduces DOX-resistance via *TUG1* downregulation. Since polydatin treatment in *TUG1*-silenced cells decreases AKT phosphorylation, inhibition of TUG1/AKT axis is required for its regulation of DOX-resistance in osteosarcoma cells [83].

Forkhead box C2 (FOXC2) is a critical transcription factor in tumor angiogenesis and MDR, functioning through EMT promotion [84]. ABCB1 plays a significant role in pumping external molecules through ATP hydrolysis that reduces the chemosensitivity of tumor cells [85]. *FOXC2-AS1* is an lncRNA that regulates FOXC2 to promote DOX resistance via ABCB1 upregulation [86]. It is involved in the regulation of intracellular Ca^2+^ levels and the activation of the Ca^2+^-FAK signaling pathway [87]. It downregulates p15 and inhibits apoptosis via recruitment of EZH2 and SU212 [88]. *FOXC2-AS1* and *FOXC2* upregulations were observed in DOX-resistant osteosarcoma tissues and cell lines. *FOXC2-AS1* is involved in *FOXC2* upregulation through the formation of a stable RNA duplex, which upregulates ABCB1 in DOX-resistant osteosarcoma cells [86]. Simultaneous high expression levels of *FOXC2-AS1* and *ABCB1* are the main reason for DOX-resistance in OS cells. Silencing *FOXC2-AS1* and *ABCB1* reduces tumor growth during doxorubicin treatment. *FOXC2-AS1* regulates the methylation of ABCB1 via PRC2, which results in ABCB1 downregulation [89].

As a ceRNAs, *OIP5-AS1* upregulates WNT-7b and triggers the WNT pathway by targeting *miR-410* [90]. It also regulates various signaling pathways, including NOTCH and PI3K/AKT [91, 92]. Significant *OIP5-AS1* upregulations were shown in DOX-resistant OS tissues and cells compared to those in normal cells and chemosensitive tumor cells. Knockdown of *OIP5-AS1* suppresses proliferation and promotes apoptosis. *OIP5-AS1* has a pivotal role in the *miR-137-3p* sponging-mediated regulation of PTN expression [93]. Fibronectin‐1 (FN1) is a pivotal glycoprotein associated with cell adhesion and motility [94]. It has a critical role in cisplatin, paclitaxel and gemcitabine responses through EMT regulation [95, 96]. Significant FN1 upregulations have been reported in DOX-resistant OS cell lines and tissues. *OIP5-AS1* regulates FN1 expression through *miR-200b-3p* sponging [97].

*SNHG12* is an lncRNA involved in the tumorigenesis of various cancers, including papillary thyroid carcinoma (PTC), GC, OS, and glioma [98–101]. It can affect the Wnt/β-catenin pathway in PTC proliferation and metastasis [99]. It can also modulate the NOTCH2 pathway, which promotes OS metastasis and growth [101]. *SNHG12* upregulates CRKL through *miR-320* targeting that results in AKT/ERK activation in GC [102]. As a member of the BCL2 protein family, MCL1 plays a pivotal role in chemoresistance and apoptosis. It has been reported that *SNHG12* decreases DOX sensitivity through *miR-320a* downregulation and MCL1 upregulation [103].

In vivo and in vitro experiments confirmed that doxorubicin-resistant OS cell lines and patients have higher expression levels of *LINC00426* than their parental counterparts. Therefore, an unfavorable prognosis and no effective response to DOX are the consequences of *LINC00426* overexpression. *LINC00426* increases DOX resistance by targeting *miR-4319* in OS cells [104].

CTA downregulation has been reported in DOX-resistant OS cells. CTA promotes apoptosis and suppresses autophagy by targeting *miR-210* in OS cells. Its downregulation correlates with poor prognosis in OS patients. CTA significantly upregulates Casp8ap2 and AIFM3 [105].

ABCB1 is one of the MDR-associated genes involved in drug efflux from tumor cells [106]. *FENDRR* is an lncRNA involved in heart development through its binding to PRC2 and TrxG/MLL complexes [107]. A significant association has been reported between *FENDRR* downregulation and DOX-resistance in OS cells. *FENDRR* downregulates ABCB1 and ABCC1. It suppresses DOX resistance and induces OS cells apoptosis [108].

### Gastric cancer

Gastric cancer (GC) remains one of the most frequent malignancies and the third leading cause of neoplasm-related death globally [109, 110]. Approximately two-thirds of patients are detected in advanced tumor stages [111, 112]. Although, there is an effective response to chemotherapy in GC patients with advanced tumors, drug resistance is also a major cause of tumor growth [113].

*HOTAIR* is a lncRNA that binds to PRC2 and the LSD1/CoREST/REST complex [114]. It also increases HOXA1 hypermethylation via DNMT1 and DNMT3b upregulations [115]. An association between *HOTAIR* upregulation and advanced stage GC tumors has been reported. *HOTAIR* increases DOX resistance, cell proliferation and migration by targeting *miR-217*, resulting in GPC5 and PTPN14 upregulations in GC cells [116].

Urothelial carcinoma associated 1 (UCA1) is a non-coding RNA that has been detected in bladder cancer for the first time [117]. It is in human endogenous retrovirus H gene family, which is highly expressed in malignant bladder cancer [117]. *UCA1* upregulation promotes cell survival in bladder cancer during treatment with cisplatin [118]. It also induces DOX resistance in breast cancer tissue [119]. Its upregulation also positively correlates with poor differentiation, high grade, and poor overall survival. Knockdown of *UCA1* inhibits tumor cell proliferation. DOX can promote apoptosis in SGC7901/DOX cells by silencing *UCA1*, and also lead to cleavage of PARP protein and BCL-2 downregulation. *UCA1* had an oncogenic role in GC via regulation of cell proliferation and DOX resistance [120].

*MiR-27b* is known as a tumor suppressor that is downregulated in GC [121, 122]. It acts as an anti-angiogenic factor through its targeting of VEGF-C in GC [122]. Significant *UCA1* upregulation has been observed in GC tissues, which was negatively correlated with *miR-27b*. Downregulation of *UCA1* induces expression of *miR-27b*, resulting in a reduction in the level of anti-apoptotic proteins such as BCL2 and promotion of apoptotic proteins such as CASP3 in gastric tumor cells [123].

Myocyte enhancer factor 2D (MEF2D) is a transcription factor that is upregulated in various cancers, such as osteosarcoma [124], leukemia [125] and GC [126]. MEF2D has a key role in tumorigenesis, promoting proliferation, invasion and metastasis via repression of cell cycle arrest proteins, apoptosis, and the induction of the VEGF and TGF-b1 signaling pathways [126, 127]. *LncR-D63785* upregulation has been reported in gastric tumor cells. Reduced *lncR-D63785* expression represses cell proliferation, invasion and metastasis. *LncR-D63785* downregulation promotes the DOX-sensitivity of GC cells to apoptosis via the miR-422a/MEF2D axis. The expression levels of KLK4, FOXG1, FOXQ1 and FOXE1 are also reduced by *miR-422a*. Positive correlations exist between the *lncR-D63785*, *miR422a* and *MEF2D* expressions in DOX-resistant GC cells [128].

*NEAT1* is a component of the paraspeckle nuclear bodies involved in the transcriptional regulation of various genes. It has an oncogenic role in various tumors, including GC [129, 130]. *NEAT1* upregulation that inhibited cell proliferation and invasion has been reported in GC. Its upregulation has also been observed in DOX-resistant GC cells [131].

*MRUL* is an lncRNA that upregulates P-gp in MDR gastric tumor cells. *MRUL* silencing significantly downregulate the Bcl-2/Bax ratio, RPS13, and RPL23 while significantly upregulating JNK1 and CPP32 in the presence of DOX. Drug-induced apoptosis increases following *MRUL* depletion in GC cells [132].

### Leukemia and lymphoma

Acute myeloid leukemia (AML) is a heterogeneous bone marrow malignancy [133]. DOX is the most commonly prescribed chemotherapeutic agent for AML treatment, but chemoresistance is a big challenge [134].

*KCNQ1OT1* is reported in vaious tumors [135, 136]. It has interactions with G9a methyltransferase and the PRC2 complex [137]. Tetraspanin3 (Tspan3) is a cell-surface protein that regulates signal transductions in cell development, growth, the immune response and tumorigenesis [138]. Significant *KCNQ1OT1* upregulation has been observed in DOX-resistant AML tissues. Its knockdown increases the DOX sensitivity and suppresses the cell proliferation and invasion of AML cells. It regulates the DOX response through *miR-193a-3p* targeting that inhibits Tspan3 [139].

Enhancer of zeste homolog 2 (EZH2) is a histone methyltransferase component of the PRC2 complex that can epigenetically methylate H3K27 to inhibit gene expression [140]. *TUG1* overexpression has been reported in DOX-resistant AML tissues and cells. Interestingly, EZH2 is recruited through *TUG1* to methylate and downregulate *miR-34a*, resulting in DOX resistance in AML cells [141].

*HOXA-AS2* is located between the HOXA3 and HOXA4 genes. It acts as an oncogenic factor in promoting cell survival, proliferation and invasion [142, 143]. It is upregulated in various types of tumors and this state significantly correlates with poor prognosis. Its overexpression has been seen in patients who received DOX. *HOXA-AS2* functions as a ceRNA of *miR-520c-3p* to upregulate S100A4, resulting in DOX-resistance of AML cells [144].

The PI3K/AKT/mTOR signaling pathway plays a pivotal role in the proliferation, differentiation and viability of hematopoietic cells [145, 146]. A correlation between *linc00239* expression and tumor cell proliferation and migration in AML cells has been observed. *Linc00239* significantly increases the DOX-resistance of KG-1 and HL-60 cells through phosphorylation of AKT and mTOR, resulting in PI3K/ATK/mTOR pathway activation [147].

Chronic myeloid leukemia (CML) is a hematological malignancy resulting from BCR-ABL fusion [148]. Although CML cases respond effectively to tyrosine kinase inhibitors and chemotherapy [149], multidrug resistance proteins such as MDR1, P-gp and ABCB1 play a vital role in chemoresistance [150–152].

HuR is a member of RBP family. It stabilizes mRNA via binding to AU-rich elements, located in the 3′-UTRs of RNA [153, 154]. An association between *FENDRR* downregulation and *MDR1* expression in DOX resistant CML cells has beenreported. *FENDRR* decreases the DOX-resistance of tumor cells by downregulating MDR1 through HuR and targeting *miR-184* in CML cells [155].

DOX is one of the common treatments for Burkitt lymphoma (BL) [156], although the majority of patients have no DOX response [157]. PI3K/AKT/mTOR is a nominated pathway in lymphoma chemoresistance. Eukaryotic translation initiation factor 4E (EIF4E) is a target of the mTOR pathway, which can affect numerous cancer phenotypes [158, 159]. *MCM3AP-AS1* reportedly increases the DOX resistance of BL cells through *miR-15a* sponging and EIF4E upregulation [160].

### Liver cancer

Hepatocellular carcinoma (HCC) is one of the leading causes of cancer-related death in the world [161, 162]. *MALAT1* is an oncogenic lncRNA that promotes tumor progression and chemoresistance through various mechanisms, such as miRNA sponging and autophagy induction [163]. It is involved in alternative splicing via regulation of SR proteins [164]. It has also critical roles in various signaling pathways, such as Hippo, PI3K-AKT, MAPK, WNT and NF-κB [165–168]. *MALAT1* upregulation has been shown in MDR-HCC cells. HIF-2a upregulates MALAT1, which subsequently targets *miR-216b* during MDR regulation in HCC cells [169].

Neuro-oncological ventral antigen 1 (Nova1) is a neuron-specific RNA-binding protein that functions as an oncogene involved in the aberrant immune response [170], the resistance of cancer cells to hypoxia-related apoptosis induction [171], and tumor progression [172]. Nova1 upregulation has been observed in Huh-7 cells, and is associated with cell proliferation, migration, invasion and poor prognosis in HCC [173].

*MALAT1* and *Nova1* upregulations have been reported for DOX-resistant hepatic tumor cells in comparison with DOX-sensitive cells. *MALAT1* upregulation correlates with tumor cell proliferation, invasion and chemoresistance through Nova1 regulation. It sponges *miR-3129-5p* in DOX-resistant cells. *MALAT1* depletion triggered DOX-resistance in HCC cells by repressing the proliferation, migration, invasion and promotion of apoptosis through the MALAT1/miR-3129-5p/Nova1 axis [174].

*NEAT1* has an important role in the integrity of paraspeckles. Its upregulation has been observed in sorafenib- and DOX-resistant HCC cells. Paraspeckles have been observed in DOX-resistant HCC cells [175].

*LncARSR* is activated by AKT to target *miR-34* and *miR-449*, which results in sunitinib resistance of renal cancer cells through AXL and c-MET upregulations [176]. Correlations have been shown between *lncARSR* upregulation and the large tumor size, advanced BCLC stage, poor prognosis, and DOX resistance of HCC cells. *LncARSR* induces DOX resistance in both in vitro and in vivo studies through PTEN targeting that activates the PI3K-AKT signaling pathway [177].

Growth arrest-specific 5 (GAS5) is an lncRNA associated with a variety of biological mechanisms, such as cell proliferation, survival and DOX resistance, via regulation of The miR-21/PTEN axis. *GAS5* upregulation in HCC cells is associated with metastasis to lymph nodes and shorter overall survival time in HCC patients. It also has a key role in DOX-resistance in both in vitro and in vivo studies. *GAS5* inhibits the expression of *miR-21*, which results in PTEN upregulation [178]. *H19* is a maternally expressed gene product that functions as a tumor suppressor or oncogene. *H19* reportedly inhibits HCC cell proliferation following sorafenib or doxorubicin treatments [179].

### Colorectal cancer

LncRNA X-inactive specific transcript (XIST) is considered the most significant regulator of X chromosome inactivation in mammals via the PRC complex [180]. It also promotes NOTCH signaling by targeting *miR-137*, which results in NOTCH-1 upregulation [181]. It has been suggested that the deregulation of *XIST* plays an important role in tumor progression and prognosis [182].

Overexpression of serum and glucocorticoid-regulated kinase 1 (SGK1; one of the AGC serine/threonine protein kinases) has been associated with proliferative activity, apoptosis, adhesion and drug-resistance in numerous types of epithelial cancer [183, 184]. There is a correlation between SGK1 and DOX-mediated apoptosis in renal cancer [185]. Downregulation of SGK1 reduces cell proliferation and migration and promotes 5-FU-mediated apoptosis induction [186]. *XIST* upregulation has been reported in DOX-resistant CRC cells. *XIST* increases DOX resistance through miR-124 sponging that results in SGK1 upregulation in CRC cells [187].

BRAF-activated noncoding RNA (BANCR) is an lncRNA involved in tumorigenesis in various cancer types, such as lung cancer, GC, thyroid cancer and osteosarcoma [188]. Chromosomal segregation 1-like (CSE1L) plays a critical role in apoptosis, survival, chromosome assembly, nuclear transportation, microvesicle formation and metastasis [189, 190]. *BANCR* and *CSE1L* overexpressions have been observed in CRC cells. Direct correlations have been found between *CSE1L* and *BANCR* expressions and the clinicopathological features of CRC. BANCR increases CSE1L expression through *miR-203* sponging in CRC tissue. There is significant *miR-203* downregulation in CRC cells in comparison with controls. *BANCR* downregulation inhibits tumor progression and promotes the sensitivity of CRC cells to DOX by modulating the miR-203/CSE1L axis [191].

The NODAL signaling pathway has a key role in the regulation of chemoresistance in cancer stem cells (CSCs) [192, 193]. NODAL signaling can be protected by GAS5, contributing to the preservation and chemoresistance of CSCs. *GAS5* is a pivotal factor in the proliferation of CSCs, and thus to tumor promotion and metastasis. It also plays a key role in drug-resistance. Knockdown of *GAS5* improves chemo-sensitivity and apoptosis in the tumor cells treated with 5-FU and DOX [194].

### Thyroid and gall bladder cancers

Thyroid cancer remains the most frequent endocrine malignancy worldwide. It has a high mortality rate [195]. Anaplastic thyroid carcinoma (ATC) is the most aggressive and recurrent type of thyroid tumor that is commonly treated with DOX [196]. However, overexpression of multidrug resistance proteins causes drug resistance in such patients [197].

Signal transducer and activator of transcription 3 (STAT3) is a transcription factor activated by cytokines and growth factors involved in inflammation, tumor cell proliferation and invasion [198–200]. INO80 is involved in DNA repair and transcription [201]. Lipoprotein receptor-related protein 6 (LRP6) is targeted by PTCSC3, resulting in repression of glioma cell proliferation via suppression of WNT signaling pathway [202]. It has been reported that PTCSC3 downregulates INO80 by targeting STAT3, which reduces the DOX-resistance of ATC [203].

Gallbladder cancer is the most aggressive cancer type observed in the biliary tract. It ranks as the fifth most frequent malignancy in digestive tracts worldwide. Many patients have poor prognosis because of diagnosis in the advanced stage due to the unclear and non-specific symptoms. Autophagy has a paradoxical role in oncogenesis. The cytoprotective role of autophagy leads to stress tolerance which enables tumor resistance toward chemotherapy [204].

Gallbladder cancer drug resistance-associated lncRNA1 (GBCDRlnc1) is a unique lncRNA mediating resistance to chemotherapy. *GBCDRlnc1* upregulation has been reported in gallbladder tumor cells. *GBCDRlnc1* maintained PGK1 stability by inhibiting its ubiquitination leading to ATG5 and ATG12 downregulations in DOX-resistant tumor cells. *GBCDRlnc1* upregulation correlates with poorer histological grade and advanced tumor stage [205].

### Prostate and urothelial cancers

PRC2 is in the methyltransferase protein family, which methylates lysine of histone H3 to suppress gene expression. The PRC2 complex is comprised of several components, including EZH1, EZH2, SUZ12 and EED [206, 207]. Significant *LINC-PINT* upregulation has been observed in clear cell renal cell carcinoma (ccRCC) cells, correlating with sex, pT and tumor stage. The *LINC-PINT* levels also negatively correlate with DFS and OS in patients. *LINC-PINT* induces cell proliferation, but represses apoptosis via EZH2 targeting in ccRCC cells. DOX upregulates *P53* and *LINC-PINT* in ccRCC tissues [208].

*GAS5* is a tumor suppressor that is downregulated in HCC, GC and ovarian cancer [209–211]. Its downregulation has also been reported in bladder transitional cell carcinoma (BTCC) tissues and cells, where it is associated with higher grades of cancer. It inhibits cell proliferation and DOX resistance in BTCC cells through downregulation of BCL-2 [212]. There are also *HOTAIR* upregulations in transitional cell carcinoma (TCC) tissues and cells and these correlate with higher histological grades, shorter overall survival, and reduced DOX sensitivity [213].

Lysyl oxidase-like 1 (LOXL1) is an extracellular matrix (ECM) protein in the the copper-dependent monoamine lysyl oxidase family, which is involved in oxidation of collagens and elastin [214]. *LOXL1-AS1* is located in the opposite strand of LOXL1 [215]. The epidermal growth factor receptor (EGFR) is a member of the receptor tyrosine kinases (RTK) family, participating in cell proliferation, differentiation and tumor progression [216, 217]. Overexpression of EGFR has been reported in a variety of tumor types [218]. It has been reported that EGFR regulates *LOXL1-AS1* expression via *miR-let-7a-5p* in prostate cancer (PCa) cells. *LOXL1-AS1* is downregulated in DOX-resistant PCa cells compared with DOX-sensitive cells. There is a significant *miR-let-7a-5p* upregulation in DOX-resistant PCa cells. *MiR-let-7a-5p* reduces the promoting role of *LOXL1-AS1* on DOX-resistant cell proliferation [219].

## Conclusions

Despite its wide clinical applications, DOX can affect the quality of life of cancer patients due to side effects during and after treatment. Clarifying the molecular basis of DOX resistance is essential for the development of novel therapeutic strategies with fewer and less impactful side effects in cancer patients. LncRNAs have critical roles in drug resistance in various tumors. In this review, we have summarized the current state of knowledge on all the lncRNAs associated with DOX resistance in various tumors. This should pave the way to introducing an lncRNA panel marker for the prediction of the DOX response among cancer patients. The majority of lncRNAs promote DOX-resistance in the various tumor types.

## Data Availability

The datasets used and/or analyzed during this study are available from the corresponding author on reasonable request.

## Abbreviations

DOX: Doxorubicin
lncRNAs: Long non-coding RNAs
EMT: Epithelial–mesenchymal transition
ADR: Adriamycin
MDR: Multidrug resistance
MRP1: Multidrug resistance protein 1
ABC: ATP-binding cassette
ANXA1: Annexin A1
PARP: Poly ADP-ribose polymerase
OS: Osteosarcoma
TUG1: Taurine upregulated gene 1
FN1: Fibronectin‐1
PTC: Papillary thyroid carcinoma
GC: Gastric carcinoma
UCA1: Urothelial carcinoma associated 1
AML: Acute myeloid leukemia
Tspan3: Tetraspanin 3
CML: Chronic myeloid leukaemia
BL: Burkitt lymphoma
EIF4E: Eukaryotic translation initiation factor 4E
HCC: Hepatocellular carcinoma
Nova1: Neuro-oncological ventral antigen 1
XIST: X-inactive specific transcript
BANCR: BRAF-activated noncoding RNA
CSE1L: Chromosomal segregation 1-like
GAS5: Growth arrest-specific 5
ATC: Anaplastic thyroid carcinoma
LRP6: Lipoprotein receptor related protein 6
GBCDRlnc1: Gallbladder cancer drug resistance-associated lncRNA1
ECM: Extracellular matrix
EGFR: Epidermal growth factor receptor
RTK: Receptor tyrosine kinases
PCa: Prostate cancer
ANLN: Anillin actin binding protein
LINP1: LncRNA in non-homologous end-joining pathway 1
FOXC2: Forkhead box C2
MEF2D: Myocyte enhancer gactor 2D
EZH2: Enhancer of zeste homolog 2
SGK1: Serum and glucocorticoid-regulated kinase 1
STAT3: Signal transducer and activator of transcription 3
LOXL1: Lysyl oxidase-like 1
BTCC: Bladder transitional cell carcinoma
TCC: Transitional cell carcinoma
